# A rare case of complicated typhoid fever presenting with secondary Hemophagocytic Lymphohistiocytosis

**DOI:** 10.1093/omcr/omae151

**Published:** 2024-12-10

**Authors:** Mir Wasim Ali, Soumadip Rakshit, Atreyee Sarkar, Md Karimulla Mondal, Anup Kumar Datta, Uttara Chatterjee

**Affiliations:** Department of Internal Medicine, IPGMER & SSKM Hospital, 242 Harish Mukherjee Road, Kolkata 700020, India; Department of Internal Medicine, IPGMER & SSKM Hospital, 242 Harish Mukherjee Road, Kolkata 700020, India; Department of Pathology, IPGMER & SSKM Hospital, 242 Harish Mukherjee Road, Kolkata 700020, India; Department of Internal Medicine, IPGMER & SSKM Hospital, 242 Harish Mukherjee Road, Kolkata 700020, India; Department of Internal Medicine, IPGMER & SSKM Hospital, 242 Harish Mukherjee Road, Kolkata 700020, India; Department of Pathology, IPGMER & SSKM Hospital, 242 Harish Mukherjee Road, Kolkata 700020, India

**Keywords:** enteric fever, hematochezia, hemophagocytic lymphohistiocytosis, rash, salmonella enterica Typhi

## Abstract

Enteric fever is one of the important causes of tropical fever with a prevalence of 11–21 million cases worldwide annually. It encompasses both typhoid and paratyphoid fever. *Salmonella enterica* Typhi is the causative organism for typhoid fever, manifesting as an uncomplicated febrile illness to life-threatening sepsis with multiorgan dysfunction. It is complicated by neuropsychiatric manifestation (2%–40%), gastrointestinal bleeding (10%), and intestinal perforation (1%–3%). Hemophagocytic Lymphohistiocytosis (HLH) is a rare complication of typhoid fever. Herein we report the case of a 24-year-old male from North-Eastern India, presenting with spikes of fever, altered behavior, a generalized rash, haematochezia, and hemophagocytes on bone marrow examination due to hemophagocytic lymphohistiocytosis secondary to complicated typhoid fever.

## Introduction

Hemophagocytic lymphohistiocytosis is a life-threatening inflammatory condition characterized by hyperactive but ineffective immune system activation. It occurs either spontaneously in genetically predisposed patients, as in primary HLH, or may be triggered by infections, malignancy, or autoimmune conditions as in secondary HLH [1]. In infections, the persistence of fever even after appropriate antibiotic therapy, accompanied by the deterioration of the patient, progressive cytopenias, hypertriglyceridemia, hyperferritinemia, hepatosplenomegaly, and rising transaminases should raise the suspicion of HLH and prompt additional tests, such as bone marrow examination. The presence of hemophagocytes in bone marrow, lymph nodes, liver, or spleen will help differentiate between secondary HLH and progression to severe sepsis. Here we present a case of HLH secondary to complicated typhoid fever nonresponsive to antimicrobials, with progressive deterioration of the patient as well as laboratory parameters. With early initiation of glucocorticoid therapy rapid recovery was obtained.

## Case report

**Figure 1:**
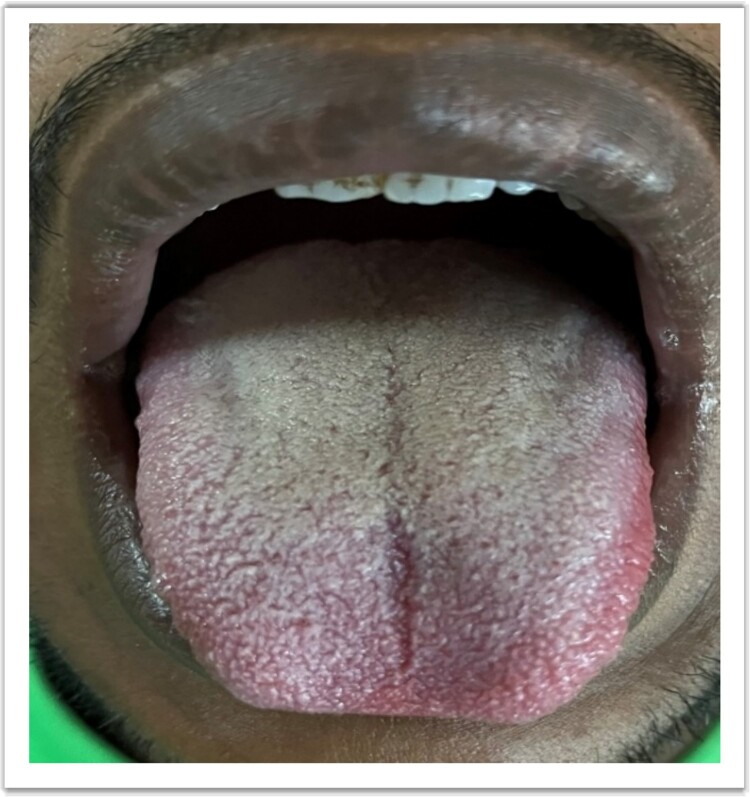
Coated tongue.

**Figure 2:**
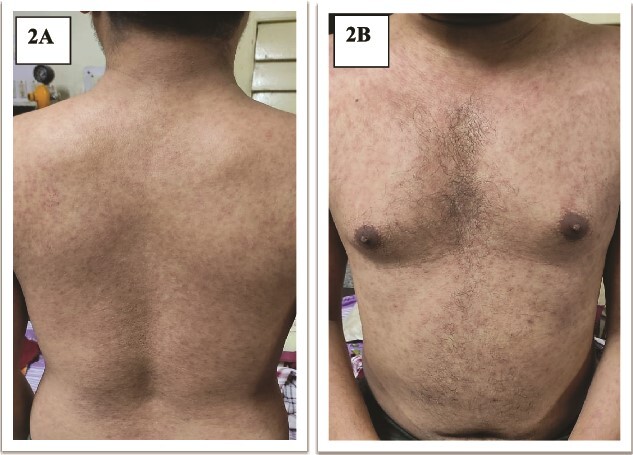
(A and B): Rose spots.

**Figure 3:**
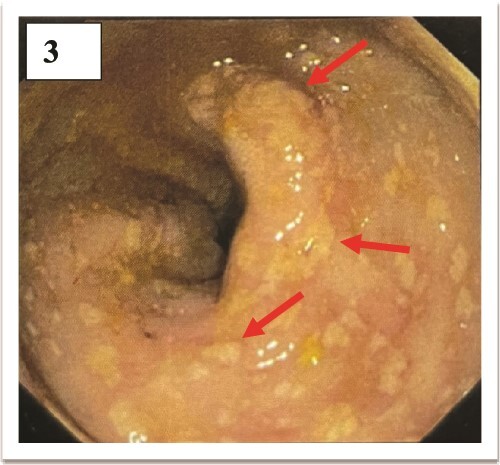
Multiple terminal ileal ulcer.

**Figure 4:**
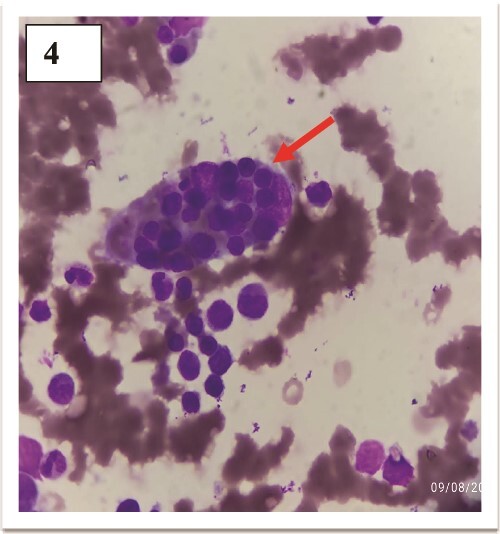
Hemophagocytes in bone marrow.

**Figure 5:**
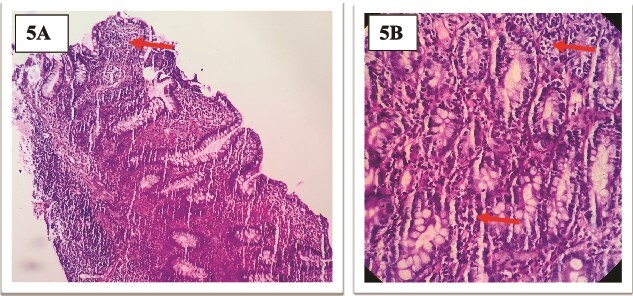
(A and B): Histopathology of terminal ileal biopsy showing dense leukocytic infiltrate of neutrophils, plasma cells and lymphocytes in lamina propria without any granuloma suggesting cryptitis with active colitis.

A 24-year-old previously healthy male, a resident of a remote village with poor sanitation presented with sudden onset high-grade intermittent 7-day history of fever with 3 spikes per day in a stepladder pattern accompanied by a confusional state and a 5-day history of left hypochondrium pain. On a general survey, blood pressure was 116/80 mmHg with tachycardia (heart rate 108/minute), without any evidence of respiratory distress. Relative bradycardia was evident during the spikes of fever. No history of rash or any gastrointestinal bleeding could be elicited. The coated tongue was visible on oral examination (Fig. 1). Hepatosplenomegaly was observed without any lymphadenopathy. The neurological survey was normal except for the confusional state. Other systemic examinations were within normal limits. During the hospital stay, the patient developed a blanchable morbilliform papular salmon-colored evanescent rash on day 8 of fever starting in the front of the chest and abdomen and gradually progressing to the back for over 1 day (Fig. 2A and B). The rash resolved spontaneously in the next 2 days. Ceftriaxone was started empirically. Meanwhile, Typhi Dot IgM came out to be reactive. Scrub typhus, leptospirosis, dengue, malaria, and kala-azar were ruled out. The hematological assessment revealed transaminitis [ALT 174 units/L (<35 units/L), AST 148 units/L (<45 units/L)] without any nephropathy. Markers of inflammation were elevated [Ferritin 380 ng/ml (5–148 ng/ml), ESR 68 mm/1^st^ hour (2–18 mm/1^st^ hour), CRP 112 mg/dl (<0.5 mg/dl)]. Normocytic normochromic anemia (Hemoglobin 100 gm/L, Total leukocyte count 4.8 × 10^9^/L, Platelet 280 × 10^9^/L) was present without other cytopenia. Empirical treatment was continued and the frequency of fever was reduced but no improvement was seen in terms of neurological complications and gastrointestinal symptoms. Blood cultures grew *Salmonella enterica* serotype Typhi after 3 days via microbroth dilution method by VITEK2. The organism was sensitive to ceftriaxone (by Kirby Bauer’s disc diffusion method). On day 5 of antibiotic therapy with ceftriaxone, the patient developed severe abdominal pain along with 3 episodes of hematochezia. An urgent colonoscopy revealed multiple terminal ileal ulcers (Fig. 3). No evidence of perforation was seen on computed tomography enterography. This time the patient started developing high-grade fever with delirium. Cerebrospinal fluid for neuroviral panel, autoimmune encephalitis profile, and Anti-Nuclear Antibody (ANA) were negative with no evidence of hypocomplementemia. The patient started developing pancytopenia (Hemoglobin 61 gm/L, WBC 3.4 × 10^9^/L, Platelet 70 × 10^9^/L). Coagulopathy was absent [INR (International Normalized Ratio)- 1.01]. Pancytopenia with low ESR (ESR 7 mm/1^st^ hour), hyperferritinemia (Ferritin 11 300 ng/ml), hypertriglyceridemia [triglyceride 410 mg/dl (<150 mg/dl)], hypofibrinogenemia [1.4 gm/L (2–4 gm/L)], and transaminitis (ALT 190 units/L, AST 379 units/L) in the background of high-grade fever with splenomegaly in a patient with typhoid infection, raised the suspicion for Hemophagocytic Lymphohistiocytosis which warranted bone marrow study. The presence of hemophagocytes in bone marrow (Fig. 4) corroborated with the diagnosis and the patient was started on parenteral dexamethasone. The patient’s general well-being improved in terms of complete remission of fever, and neurological and gastrointestinal complications. Terminal ileal biopsy sample was negative for tissue CBNAAT (Cartridge Based Nucleic Acid Amplification Test), and histopathology revealed cryptitis consisting of lymphocytes, and plasma cells along with few polymorphs in lamina propria, without any granuloma suggesting active colitis (Fig. 5A and B). Upper gastrointestinal endoscopy was also normal. Antibiotics were discontinued after a total duration of 2 weeks and dexamethasone was tapered off over the next week.

## Discussion

Typhoid fever is one of the important causes of tropical fever caused by *S. enterica* subspecies Typhi. This gram-negative bacillus has the propensity to cause a wide array of illnesses carrying significant morbidity as well as mortality, thus requiring frequent hospitalization even in the 21^st^ century [2, 3]. The possibility of typhoid fever should be considered in a febrile patient with fever for more than 3 days with accompanying gastrointestinal symptoms, living in or traveling from an endemic area, like India. Approximately 10%–15% of patients develop complications including Neuropsychiatric and Gastro-Intestinal bleeding as seen in this case [4]. Hemophagocytic lymphohistiocytosis (HLH) is one of such rare but serious complications of typhoid fever. Infection-associated HLH (IAH) has been most commonly associated with viral infections, especially Epstein–Barr virus (EBV), but has also been reported with pyogenic infections like Salmonellosis [5, 6]. The diagnosis of HLH secondary to typhoid fever is challenging. Fever, hepatitis, and cytopenia in the background of splenomegaly may be primary manifestations of *Salmonella typhi* infection as well as sepsis [7, 8]. However, persistent fever despite appropriate antimicrobial administration, along with cytopenias, and rising liver enzymes, in the background of splenomegaly, should raise an early suspicion for HLH, as a late or missed diagnosis of HLH carries a high mortality risk [9]. Cytokine storm by persistently activated lymphocytes and histiocytes in response to typhoid bacilli is the key mechanism responsible for such high mortality and warrants prompt treatment. On the other hand, the initial proliferation of the bacilli in the Peyer’s patches, dissemination to the liver, spleen, and reticuloendothelial system, and finally release into the bile and re-infection of the ileal lymphoid tissue creates the classical bowel pathology associated with *S. Typhi* infection. Although most of the cases of *Salmonella* gastroenteritis are self-limited having a brief course, severe Salmonella enteritis with delayed gastrointestinal bleeding, is a rare complication of typhoid fever. Treatment of the underlying infection and parenteral glucocorticoid, are sufficient to control the cytokine storm in sixty to seventy percent of patients with IAH. But for severe cases with deteriorating cardiovascular, pulmonary, renal, hepatic, or neurologic function and not responding to glucocorticoids, induction chemotherapy with eight weeks of Etoposide and Dexamethasone is considered as per HLH-94 protocol [10]. In rare instances of massive lower gastrointestinal bleeding, successful use of selective angiography and platinum coil embolization is associated with higher survival rates and better outcomes.

## Conclusion

In patients with tropical fever like enteric fever, with progressive worsening of the patient, despite receiving appropriate treatment, a high degree of clinical suspicion for Hemophagocytic Lymphohistiocytosis should be kept in mind for the physician to initiate prompt treatment as delayed diagnosis is associated with higher mortality.

## References

[1] Kim YR, Kim DY. Current status of the diagnosis and treatment of hemophagocytic lymphohistiocytosis in adults. Blood Res 2021;56:S17–25. 33935031 10.5045/br.2021.2020323 PMC8094004

[2] Malik AS . Complications of bacteriologically confirmed typhoid fever in children. J Trop Pediatr 2002;48:102–8. 12022423 10.1093/tropej/48.2.102

[3] Ochiai RL, Acosta CJ, Danovaro-Holliday M. et al. A study of typhoid fever in five Asian countries: disease burden and implications for controls. Bull World Health Organ 2008;86:260–8. 18438514 10.2471/BLT.06.039818 PMC2647431

[4] Devaranavadagi RA, Srinivasa S. A study on clinical profile of typhoid fever in children. Int J Contemp Pediatr 2017;4:1067–73.

[5] Rouphael NG, Talati NJ, Vaughan C. et al. Infections associated with haemophagocytic syndrome. Lancet 2007;7:814–22. 10.1016/S1473-3099(07)70290-6 PMC7185531 18045564

[6] Singh ZN, Rakheja D, Yadav TP. et al. Infection-associated haemophagocytosis: the tropical spectrum. Clin Lab Haematol 2005;27:312–5. 16178911 10.1111/j.1365-2257.2005.00717.x

[7] Morimoto A, Nakazawa Y, Ishii E. Hemophagocytic lymphohistiocytosis: pathogenesis, diagnosis, and management. Pediatr Int 2016;58:817–25. 27289085 10.1111/ped.13064

[8] Çaksen H, Akbayram S, Öner AF. et al. A case of typhoid fever associated with hemophagocytic syndrome. J Emerg Med 2003;25:321–2. 14585463 10.1016/s0736-4679(03)00212-9

[9] Ray U, Dutta S, Bandyopadhyay S. et al. Infections and HLH - experience from a tertiary care center. J Assoc Physicians India 2019;67:54–7. 30935175

[10] Henter JI, Aricò M, Egeler RM. et al. HLH-94: a treatment protocol for hemophagocytic lymphohistiocytosis. HLH study Group of the Histiocyte Society. Med Pediatr Oncol 1997;28:342–7. 9121398 10.1002/(sici)1096-911x(199705)28:5<342::aid-mpo3>3.0.co;2-h

